# Complete Genome Sequence of *Vibrio gazogenes* ATCC 43942

**DOI:** 10.1128/genomeA.00733-17

**Published:** 2017-07-27

**Authors:** Phani M. Gummadidala, Michael E. Holder, Jacqueline L. O’Brien, Nadim J. Ajami, Joseph F. Petrosino, Chandrani Mitra, Yung Pin Chen, Alan W. Decho, Anindya Chanda

**Affiliations:** a Environmental Health Sciences, University of South Carolina, Columbia, South Carolina, USA; b The Alkek Center for Metagenomics and Microbiome Research, Department of Molecular Virology and Microbiology, Baylor College of Medicine, Houston, Texas, USA

## Abstract

*Vibrio gazogenes* ATCC 43942 has the potential to synthesize a plethora of metabolites which are of clinical and agricultural significance in response to environmental triggers. The complete genomic sequence of *Vibrio gazogenes* ATCC 43942 is reported herein, contributing to the knowledge base of strains in the *Vibrio* genus.

## GENOME ANNOUNCEMENT

*Vibrio gazogenes* is an estuarine Gram-negative bacterium that is known for its ability to synthesize industrially relevant proteins, such as amylases and proteases (1), and bactericidal and fungicidal pigments, magnesidin A (2), prodigiosins, and cycloprodigiosins (3).

*V. gazogenes* ATCC 43942 was recently studied by our laboratory for its response to aflatoxin, a hepatocarcinogen and a mycotoxin that is produced from a group of filamentous fungi under the genus *Aspergillus*. The bacterium demonstrated the ability to generate a group of metabolites (named aflatoxin response metabolites [ARMs]) that were able to inhibit aflatoxin synthesis in the aflatoxin producer *Aspergillus parasiticus* (4). Also, in our ongoing (unpublished data) studies, we have consistently observed the ability of this *Vibrio* strain to degrade mycotoxins and generate a unique set of antibiotics that are active against multiple-antibiotic-resistant bacterial strains. These observations prompted us to categorize this bacterium as clinically and agriculturally significant and have provided the rationale for sequencing its genome.

Genomic DNA extraction (10 to 20 μg) was performed using the PureLink genomic DNA minikit (Invitrogen). The extracted DNA was quantified using a NanoDrop 1000 (Thermo Scientific), and the quality of the DNA was assessed by running a 1% agarose gel with the DNA gel stain SYBR safe (Life Technologies, Inc.) and visualized in a ChemiDoc MP system (Bio-Rad). DNA sequencing was performed on the Pacific Biosciences RSII platform. A single-molecule real-time (SMRT) cell, yielding 73,434 postfiltered polymerase reads and having an *N*_50_ read length of 26,245 bases and a mean read length of 16,358 bases, was used for assembly in Pacific Biosciences’s SMRT Analysis version 2.3.0 package using the RS_HGAP_Assembly.2 protocol (NCBI Prokaryotic Annotation Pipeline, accessed from https://www.ncbi.nlm.nih.gov/genome/annotation_prok). Quiver was subsequently used to polish the assembly. The finished genomic sequences were annotated with NCBI’s Prokaryotic Genome Annotation Pipeline. A high-quality finished version of the *V. gazogenes* genome is reported here as two circular chromosomes and one circular plasmid, with a mean coverage of 185×, with features as follows: (i) chromosome 1 (denoted as Chr_1), size, 3,471,064 bp; GC content, 45.5%; 2,988 proteins; 25 rRNAs; 87 tRNAs; 4 noncoding RNAs (ncRNAs); 3,153 genes; and 49 pseudogenes; (ii) chromosome 2 (denoted as Chr_2), size, 1,303,572 bp; GC content, 44.9%; 1,102 proteins; 4 tRNAs; 1,138 genes; and 32 pseudogenes; and (iii) a plasmid (denoted as P_1), size, 11,916 bp; GC content, 45.2%; 22 proteins; 23 genes; and 1 pseudogene.

The utility of prodigiosins that are synthesized by *V. gazogenes* ATCC 43942, coupled with its ability to produce unique antibiotics and mycotoxin inhibitors under custom-designed environmental settings makes this strain “chemically gifted.” In this context, its finished genomic sequence provides a necessary point of comparison with other *V. gazogenes* strains and bacterial species within the *Vibrio* genus for elucidation of the molecular factors that govern its unique metabolic profile.

### Accession number(s).

The sequence of *V. gazogenes* ATCC 43942 has been deposited in NCBI GenBank under the accession numbers CP018835, CP018836, and CP018837.
